# Supplementary material for: Characterization of the Gene Expression Profile Response to Drought Stress in Populus ussuriensis Using PacBio SMRT and Illumina Sequencing
Source: Int J Mol Sci. 2022 Mar 30;23(7):3840. doi: 10.3390/ijms23073840 (PMC8998571; doi:10.3390/ijms23073840)
Supplement: Supplementary file 1 [file ijms-23-03840-s001.zip › Figures S1-S6 containg caption.pdf]

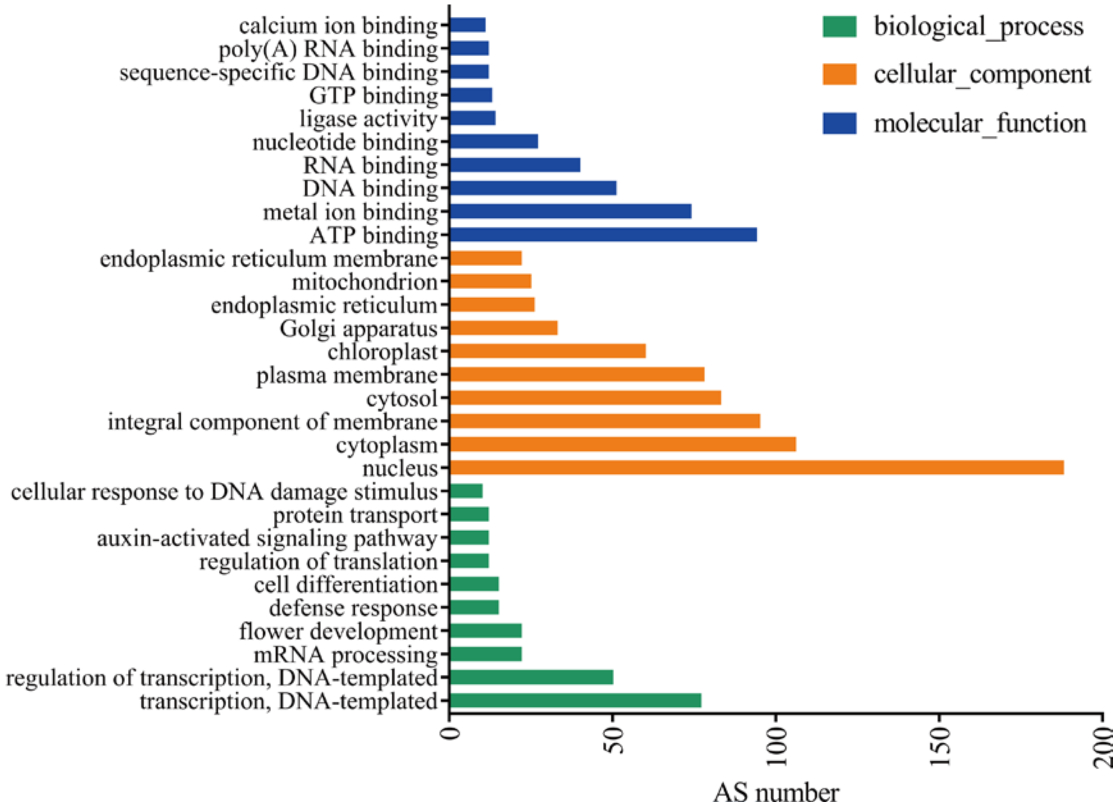

Figure S1. Gene ontology classification of alternative splicing (AS).

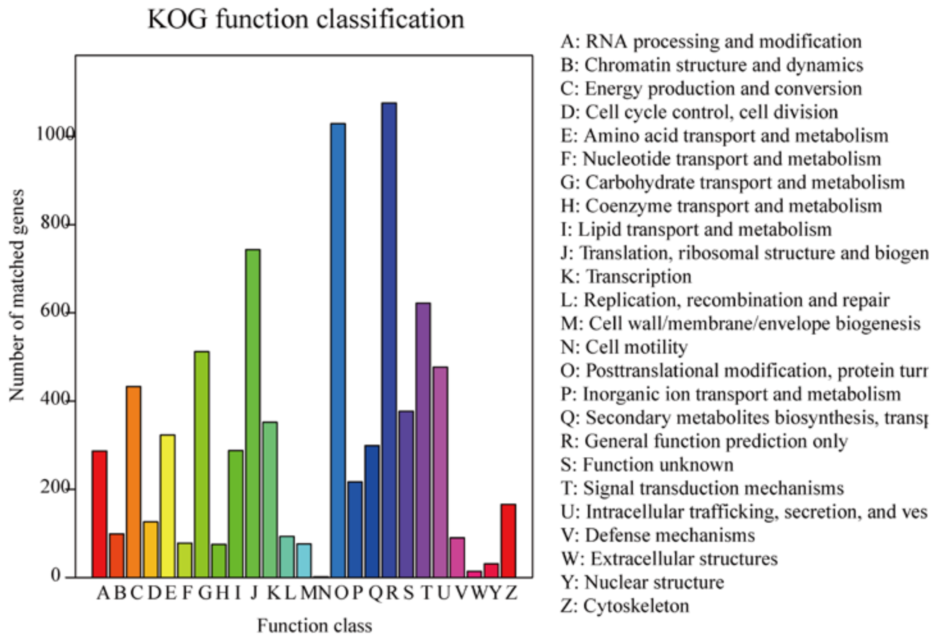

Figure S2. The euKaryotic Ortholog Groups annotation.

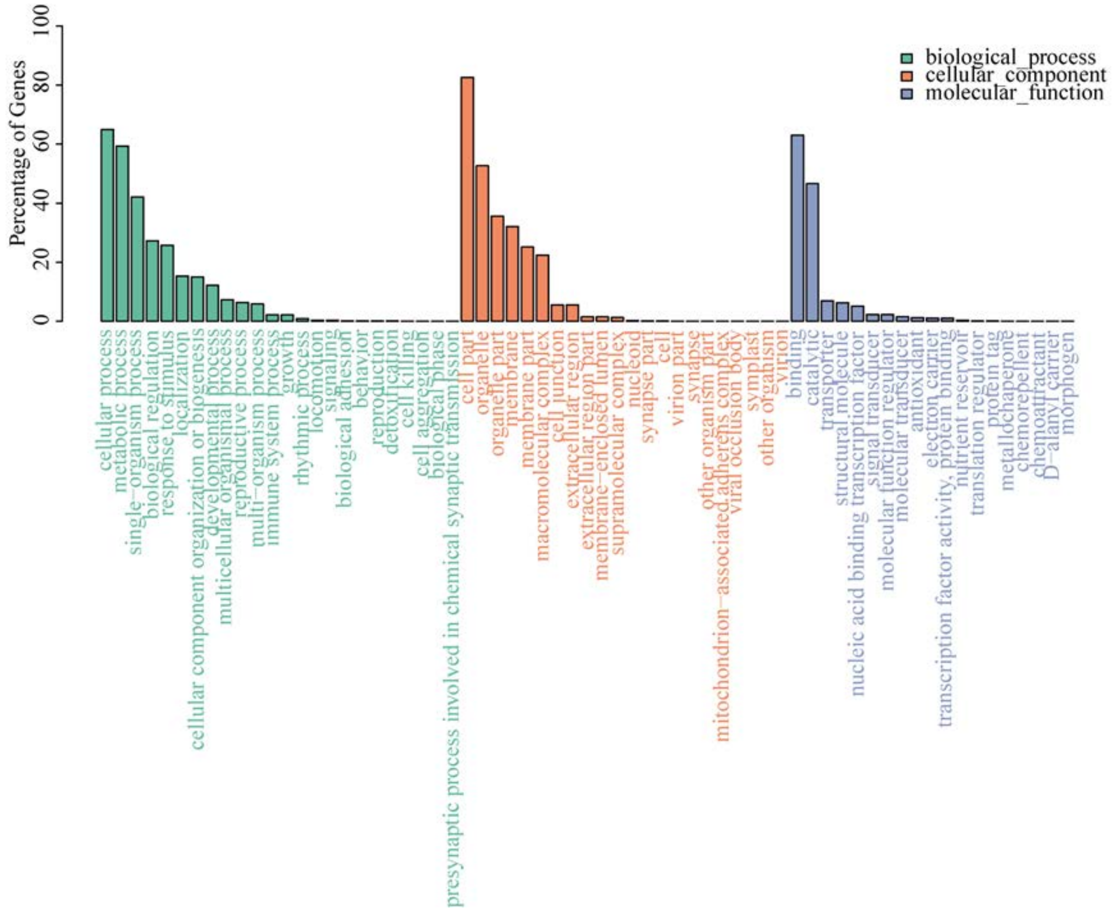

Figure S3. Gene ontology classification of all genes.

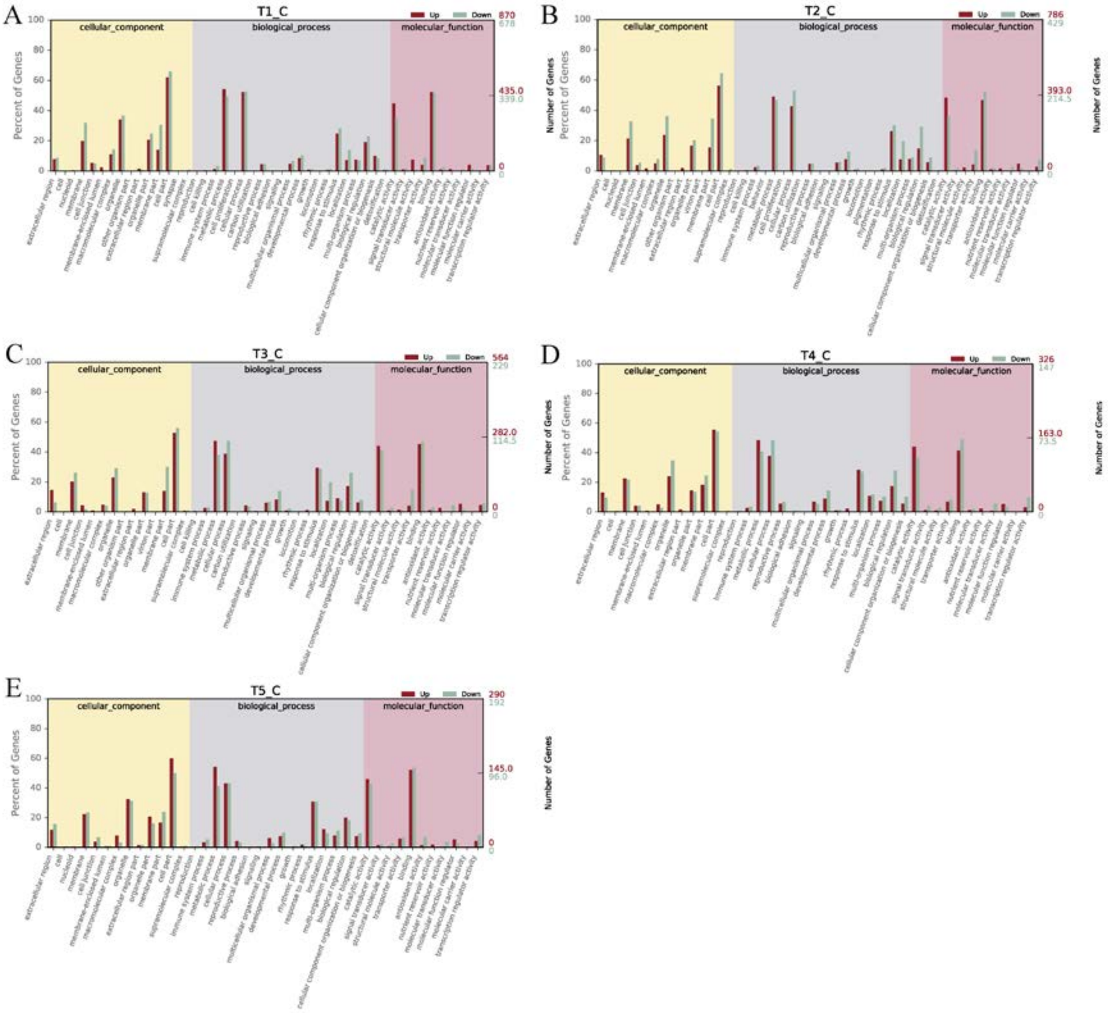

Figure S4. Gene ontology classification of differentially expressed genes.

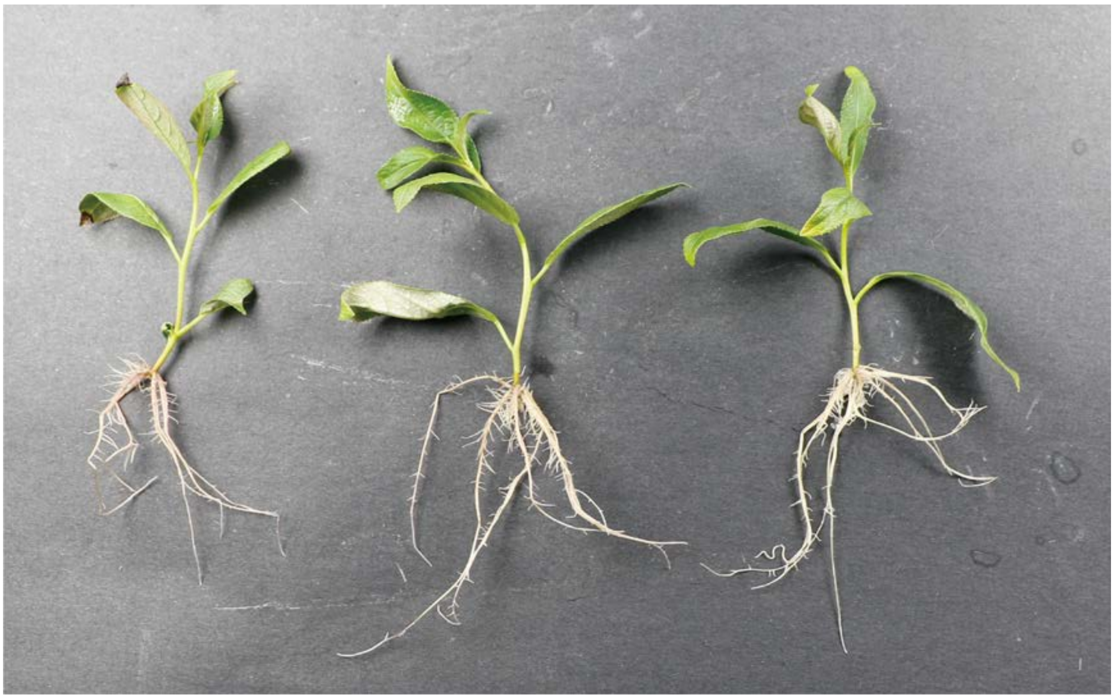

Figure S5. Three-week-old Populus ussuriensis seedlings.

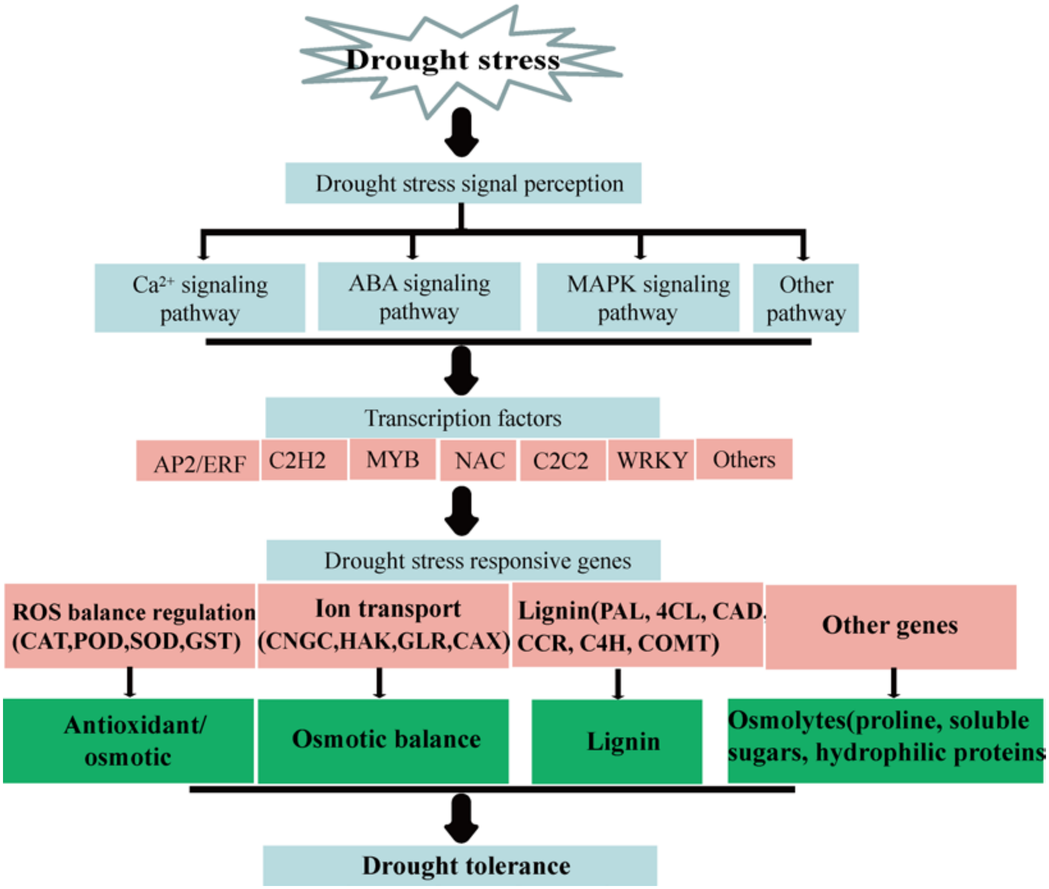

Figure S6. A hypothetical model of drought responses in *Populus ussuriensis*.
