# Supplementary material for: Characterization of the Gene Expression Profile Response to Drought Stress in Populus ussuriensis Using PacBio SMRT and Illumina Sequencing
Source: Int J Mol Sci. 2022 Mar 30;23(7):3840. doi: 10.3390/ijms23073840 (PMC8998571; doi:10.3390/ijms23073840)
Supplement: Supplementary file 1 [file ijms-23-03840-s001.zip › Table S1.pdf]

Table S1. Primers for quantitative PCR in *Populus ussuriensis.*

Gene_id	Function annotated	Primer orientation	Primer sequence
f2p30_1011_11035	CML38	forward	GTCACACTCAGTTTCATCCTC
		reverse	CTGCGTGGTTATTGTCGGGT
f5p60_1300_26955	MAPKKK18	forward	GGAGAATTTAACAAGCAAATTG
		reverse	GGACCAAGACCAGGCTCTCC
f2p21_2499_2742	NCED3	forward	CGAGACACTCCCTCTCCTGTTG
		reverse	GGGTAGAGGGTGTTGACGTTC
f7p60_2856_19557	KT2	forward	AGGGTGGTACTTTTGCATTG
		reverse	GTGGAGAGTGATTCATCTGC
f2p60_2293_3245	NCL	forward	GTGTAGGAGGTGGCACTCCTG
		reverse	GACTCCCCCATCACTCTCTC
f3p60_1980_22126	CAX7	forward	CCAATGAGCCAGAAGTCACC
		reverse	CCCAATATTAGACTTGATGTAC
f2p60_1369_7946	RAB18	forward	CCATTTCAAAGAGAGAAAAAAG
		reverse	GTCTCACCCTTGCGCTCATC
f2p37_1282_27056	LEA29	forward	CGTTCTTCAACCCCGACAGAAG
		reverse	CATTCGGCCCATGACTCGGTGG
f2p60_820_32228	DNJ11	forward	GTATCGTTAGTGTATTTTCAATC
		reverse	GGAAGCGAATGAGGCTGATTGG
f2p59_1311_26810	PER4	forward	GTAACCTTGGCCATCCTAG
		reverse	CTGCTGAAAAGAGATGTGGGC
f2p60_894_312621	GSTXC	forward	GAAGTGGTTCTACTGGATTTTG
		reverse	GCTCAAGTCCTCCACCTGAG
f2p28_837_12827	SODC	forward	GTGCTCTGAGAACAATGGTG
		reverse	GCTTAAGACCAGAAAGGTTTC
f8p60_1108_28852	ERF017	forward	GGTGGGCAGTCACTTAGTTCG
		reverse	GATTCACCTCTCATAGCAGTC
f2p24_969_11433	ZAT11	forward	GAAGAGAGATAGAGAACAGGC
		reverse	CGGTGCTGCTGCTGCTGCTG
f2p60_1403_7712	HB7	forward	GACTACAGCATGCTACGAGC
		reverse	CACTCTCCTTCCTCTACCGG
f3p60_1224_9210	MYB58	forward	CTTGCAATCTAATGAAGTTC
		reverse	CCAATATTATATGCATCTCC
f7p60_1432_25632	NAC35	forward	CATCCAACAGATGATGAGTTGG
		reverse	CTTGTAAAGATCAATCTCAGC
f2p37_1325_26690	WRKY75	forward	CACCATCATCACTAAACATG
		reverse	GCATCCAAATGTTGAAACCTAC

f2p60_1764_23398	UBQ10	forward	GGAGTCAACCCTTCACTTGGTGC
		reverse	GAGGACAAGGTGAAGGGTGGACTCC
