# Supplementary material for: Characterization of the Gene Expression Profile Response to Drought Stress in Populus ussuriensis Using PacBio SMRT and Illumina Sequencing
Source: Int J Mol Sci. 2022 Mar 30;23(7):3840. doi: 10.3390/ijms23073840 (PMC8998571; doi:10.3390/ijms23073840)
Supplement: Supplementary file 1 [file ijms-23-03840-s001.zip › Table S2.pdf]

Table S2. Overview of the RNA sequencing in *Populus ussuriensis.*

Sample	Raw Reads (Mb)	Clean Reads (Mb)	Clean Bases (Gb)	Raw Reads Q30 Rate (%)	Clean Reads Q30 Rate (%)	Mapped Reads (Mb)	Mapping Rate (%)	MultiMap Reads	MultiMap Rate (%)
CK-1	48,208,730	45,944,066	6,891,609,900	93.68	94.06	38,310,710	69.2	16,044,354	29
CK-2	48,830,500	47,039,462	7,055,919,300	93.15	93.57	35,657,838	69	14,657,924	28.4
CK-3	48,375,674	46,727,552	7,009,132,800	93.27	93.65	37,126,730	69	15,274,430	28.4
6h-1	49,867,122	47,883,300	7,182,495,000	93.64	94.02	42,069,514	69	17,311,578	28.4
6h-2	44,300,200	42,766,150	6,414,922,500	93.75	94.13	28,971,568	67.7	11,726,844	27.4
6h-3	45,803,258	44,312,524	6,646,878,600	93.81	94.17	30,016,678	67.7	11,958,736	27
12h-1	47,748,878	46,123,956	6,918,593,400	93.45	93.9	34,486,470	69.5	14,351,696	28.9
12h-2	48,434,624	46,612,778	6,991,916,700	93.08	93.52	35,768,146	70.1	14,822,522	29.1
12h-3	47,057,384	45,601,728	6,840,259,200	93.58	94.02	39,586,310	69.6	16,183,546	28.5
24h-1	46,949,778	45,693,072	6,853,960,800	93.57	94	38,723,896	69.2	16,165,064	28.9
24h-2	43,221,040	41,994,706	6,299,205,900	93.43	93.86	29,422,946	70.1	12,379,056	29.5
24h-3	47,272,212	45,457,082	6,818,562,300	93.45	93.83	40,184,286	68.8	16,574,466	28.4
48h-1	48,535,886	47,402,184	7,110,327,600	93.24	93.61	33,966,164	69.4	14,152,508	28.9
48h-2	45,470,182	44,043,960	6,606,594,000	93.67	94.04	31,610,744	70	13,251,100	29.1
48h-3	47,702,118	46,572,610	6,985,891,500	93.64	94.02	35,259,468	69.8	14,830,386	29.4
120h-1	45,605,356	44,001,044	6,600,156,600	93.94	94.28	34,757,258	69.9	14,718,010	29.6
120h-2	43,465,462	41,885,968	6,282,895,200	94.35	94.66	29,328,332	70	12,377,788	29.6
120h-3	48,166,758	46,727,276	7,009,091,400	93.95	94.33	36,410,966	69.9	15,255,106	29.3
