# Supplementary figures and images for: Characterization of the Gene Expression Profile Response to Drought Stress in Populus ussuriensis Using PacBio SMRT and Illumina Sequencing
Source: Int J Mol Sci. 2022 Mar 30;23(7):3840. doi: 10.3390/ijms23073840 (PMC8998571; doi:10.3390/ijms23073840)

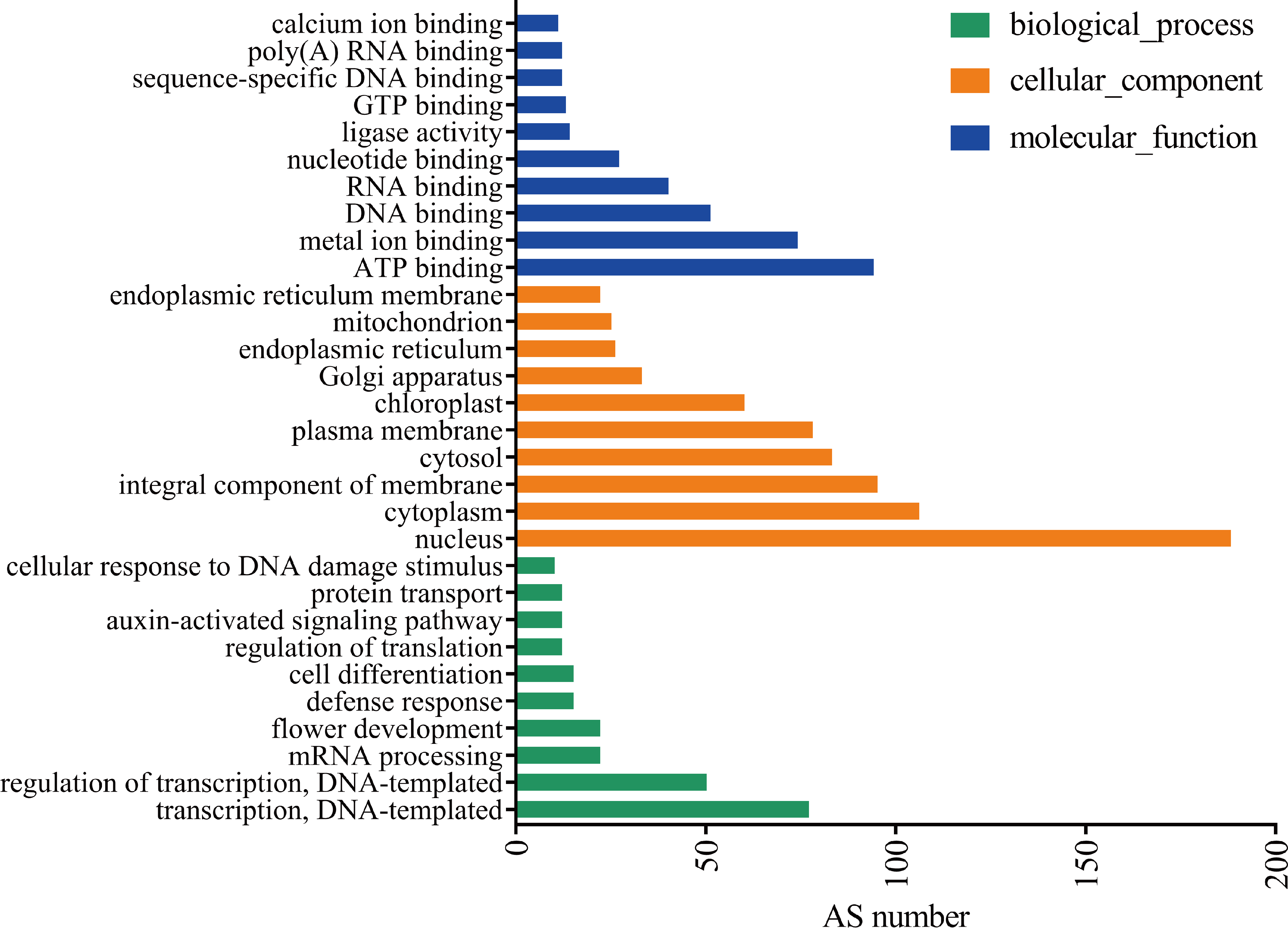

Supplement: Supplementary file 1 [file ijms-23-03840-s001.zip › Figure S1.tif]

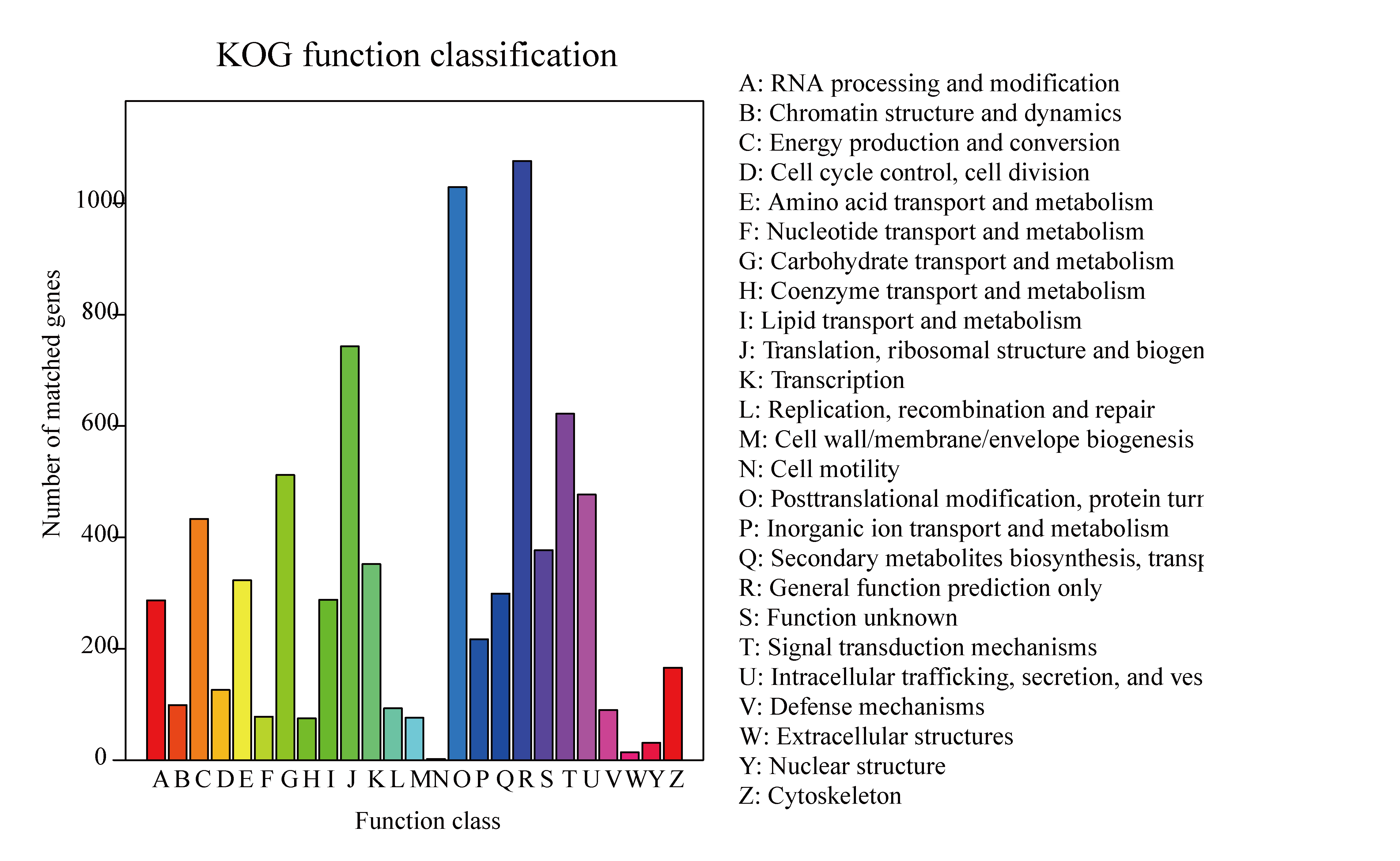

Supplement: Supplementary file 1 [file ijms-23-03840-s001.zip › Figure S2.tif]

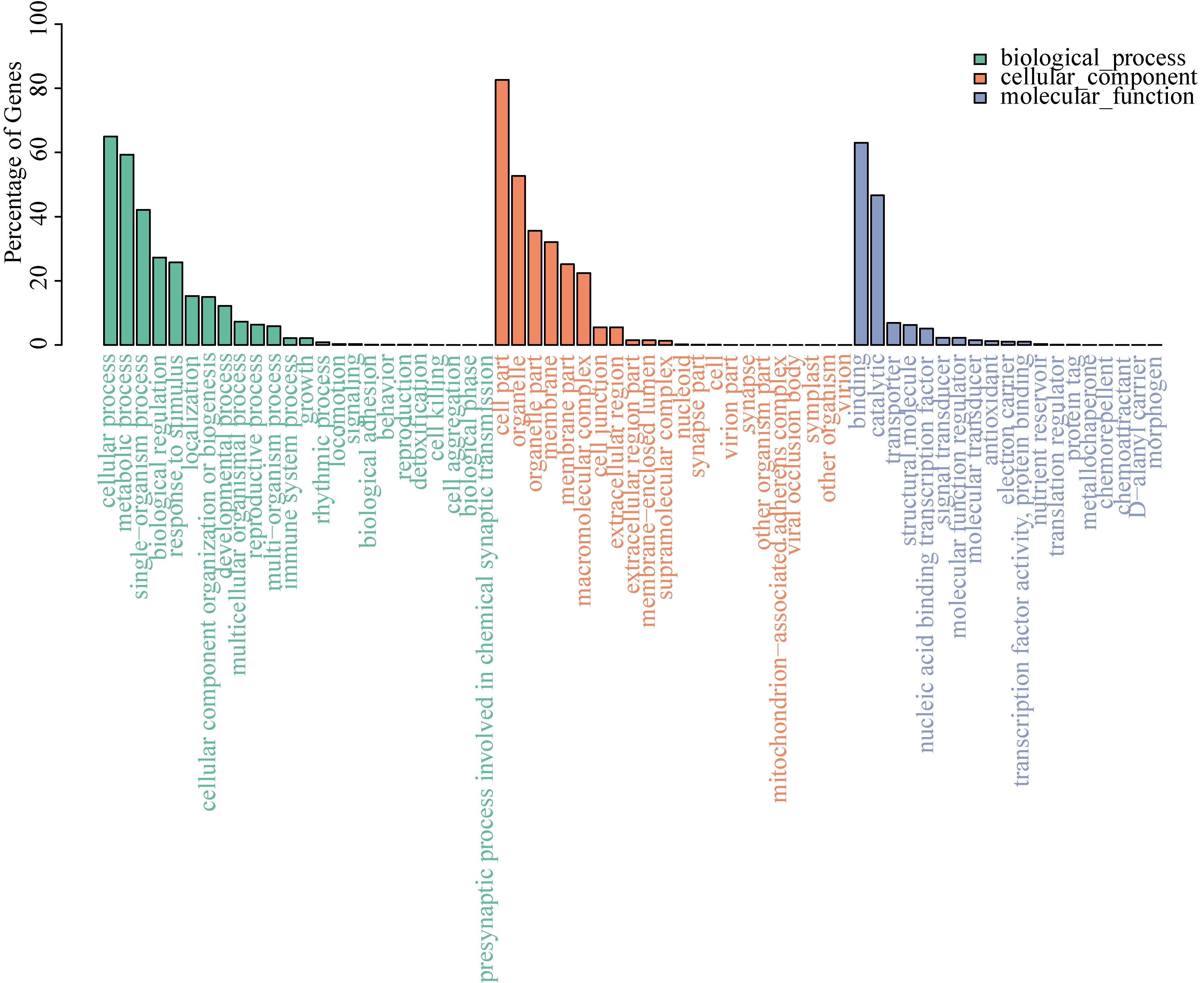

Supplement: Supplementary file 1 [file ijms-23-03840-s001.zip › Figure S3.tif]

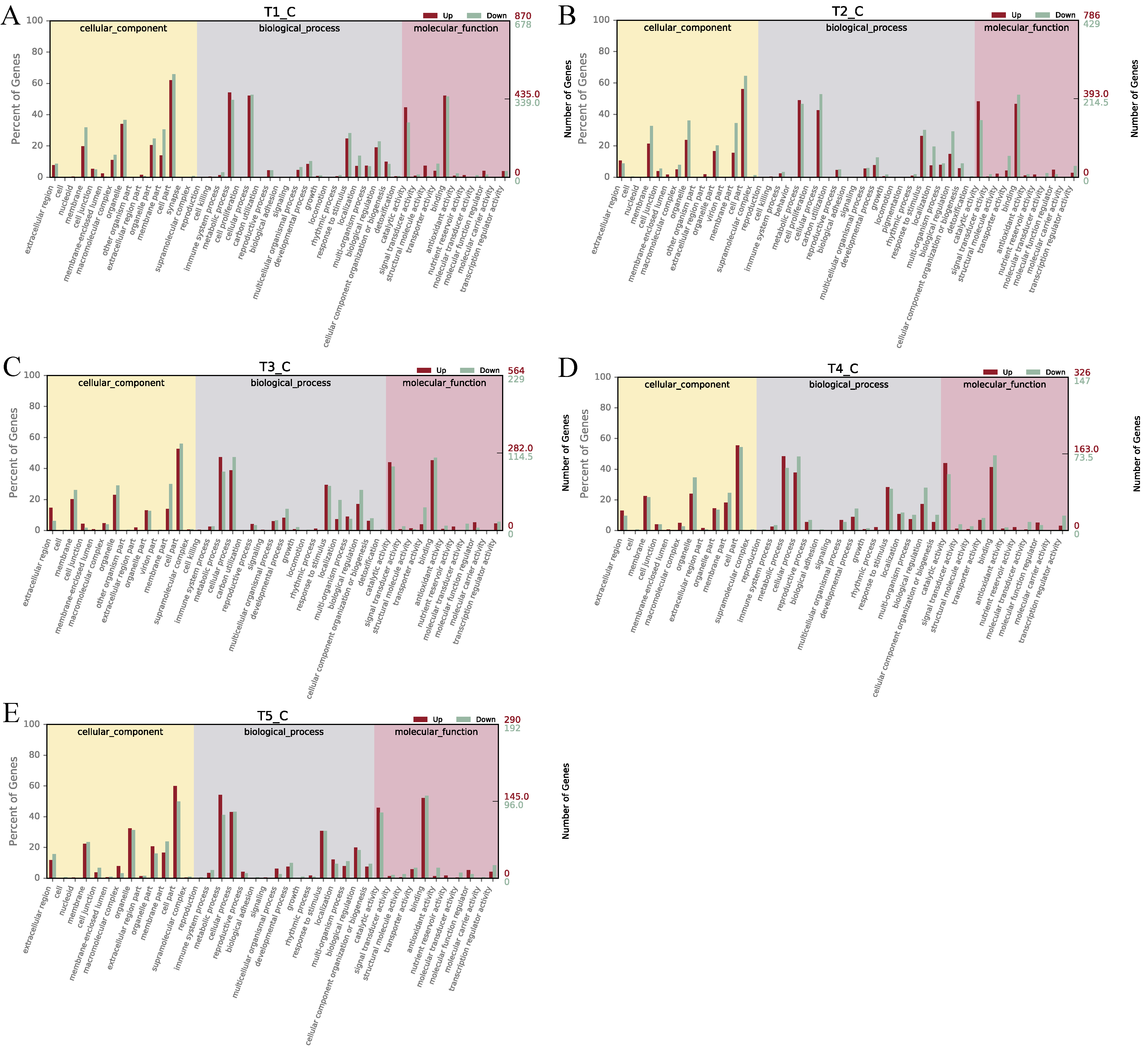

Supplement: Supplementary file 1 [file ijms-23-03840-s001.zip › Figure S4.tif]

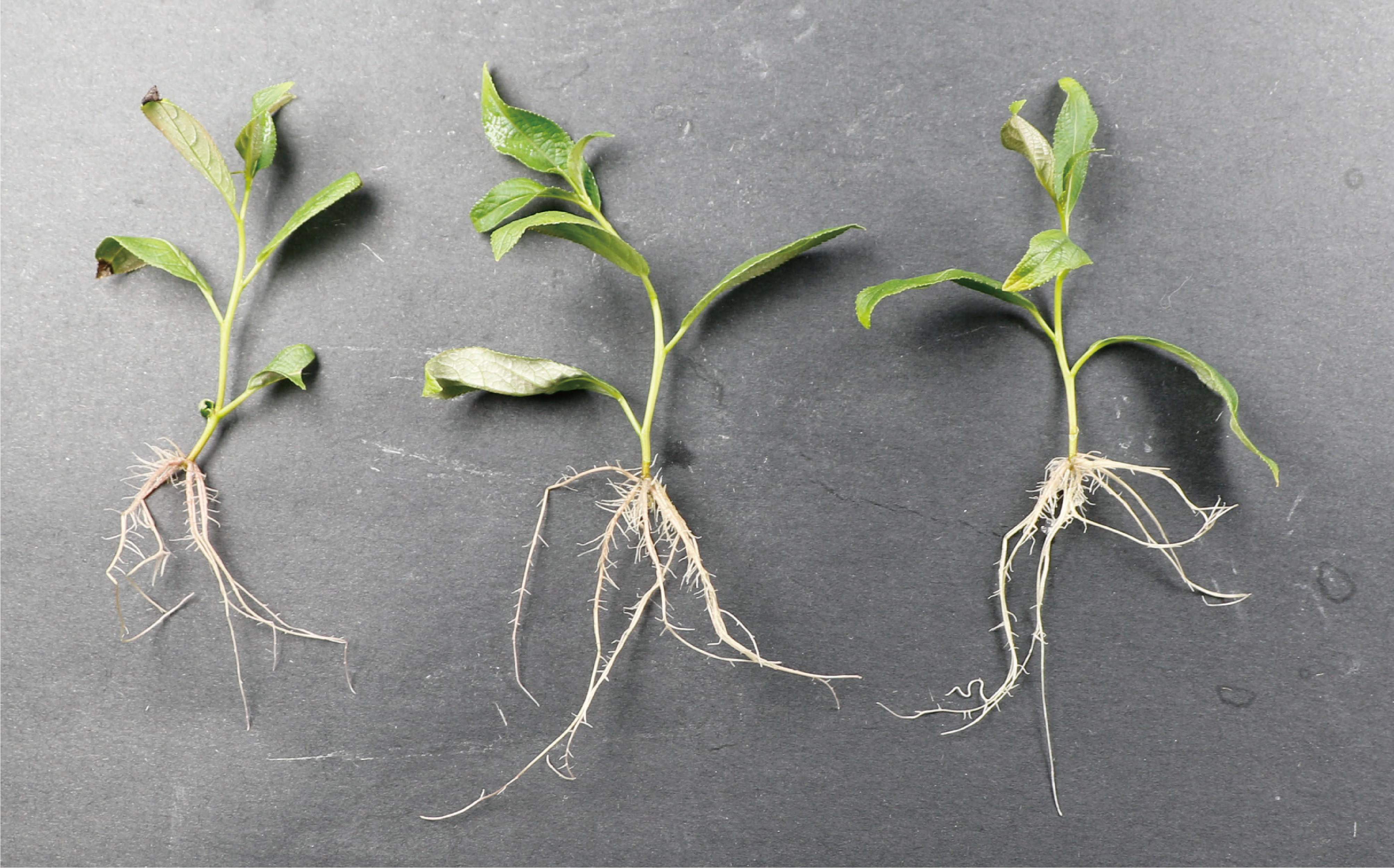

Supplement: Supplementary file 1 [file ijms-23-03840-s001.zip › Figure S5.tif]

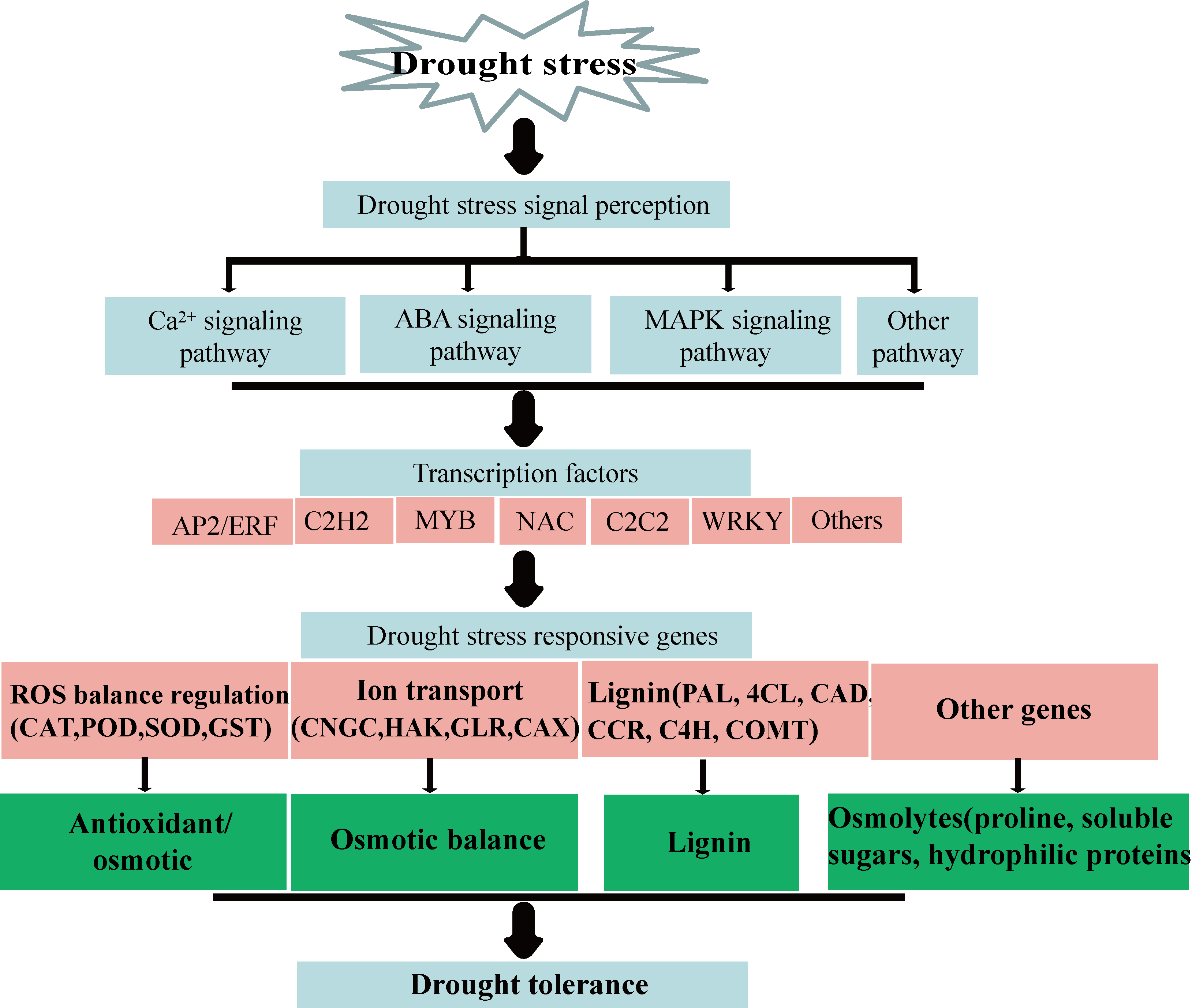

Supplement: Supplementary file 1 [file ijms-23-03840-s001.zip › Figure S6.tif]
